# Sex differences in traumatic brain injury: a multi-dimensional exploration in genes, hormones, cells, individuals, and society

**DOI:** 10.1186/s41016-019-0173-8

**Published:** 2019-10-04

**Authors:** Cheng Ma, Xin Wu, Xiaotian Shen, Yanbo Yang, Zhouqing Chen, Xiaoou Sun, Zhong Wang

**Affiliations:** 1 Department of Neurosurgery & Brain and Nerve Research Laboratory, The First Affiliated Hospital of Soochow University, Jiangsu Province, 188 Shizi Street, Suzhou, 215006 China; 2 Shanghai Medical College, Fudan University, Shanghai, 200032 China

**Keywords:** Traumatic brain injury, Gender, Sex differences, Female steroids, Microglia, Cognitive impairment, Dopamine, Social impairment

## Abstract

Traumatic brain injury (TBI) is exceptionally prevalent in society and often imposes a massive burden on patients’ families and poor prognosis. The evidence reviewed here suggests that gender can influence clinical outcomes of TBI in many aspects, ranges from patients’ mortality and short-term outcome to their long-term outcome, as well as the incidence of cognitive impairment. We mainly focused on the causes and mechanisms underlying the differences between male and female after TBI, from both biological and sociological views. As it turns out that multiple factors contribute to the gender differences after TBI, not merely the perspective of gender and sex hormones. Centered on this, we discussed how female steroid hormones exert neuroprotective effects through the anti-inflammatory and antioxidant mechanism, along with the cognitive impairment and the social integration problems it caused. As to the treatment, both instant and long-term treatment of TBI requires adjustments according to gender. A further study with more focus on this topic is therefore suggested to provide better treatment options for these patients.

## Main text

When an external force suddenly acts on the brain, the brain is severely traumatized in a moment, causing a series of physiological and psychological damage. Previous survey shows that there are 150–315 people suffering from traumatic brain injury (TBI) in every 100,000 people [1], and this ratio is still increasing with the development of transportation, especially in developing countries [2]. In 2014, TBI accounted for nearly 2.87 million emergency visits, 288,000 hospitalizations, and more than 56,800 deaths in the USA [3]. Thus, TBI has becoming a social and economic burden and poses a challenge to public health simultaneously because of the high incidence, mortality, and disability rate, as well as the grand expense of rehabilitation.

Multiple researches have revealed sex differences in TBI. They noted that the number of male patients is higher than female [4–6], due to the increased probability of injury in males. What is more, men and women are different in growing environment, neurodevelopment, and sociological attributes [7], which is hard to neglect and may also contribute to the difference [8–10].

Gender-related physiological differences have been confirmed in animal experiments, and the results of clinical trials of TBI and gender differences are still unclear. Most of them focus on the different hormone levels caused different reproductive structures. When TBI happens, estrogen and progesterone can play various neuroprotective functions such as reduce intracranial pressure (ICP), improve cerebral perfusion pressure (CPP), and neurological score [11]. However, the neuroprotective mechanisms of estrogen and progesterone are still controversial and need further research. Also, researchers begin to focus on the role of microglia, which previously focused on male subjects, but started to shift to the other gender recently.

**Fig. 1:**
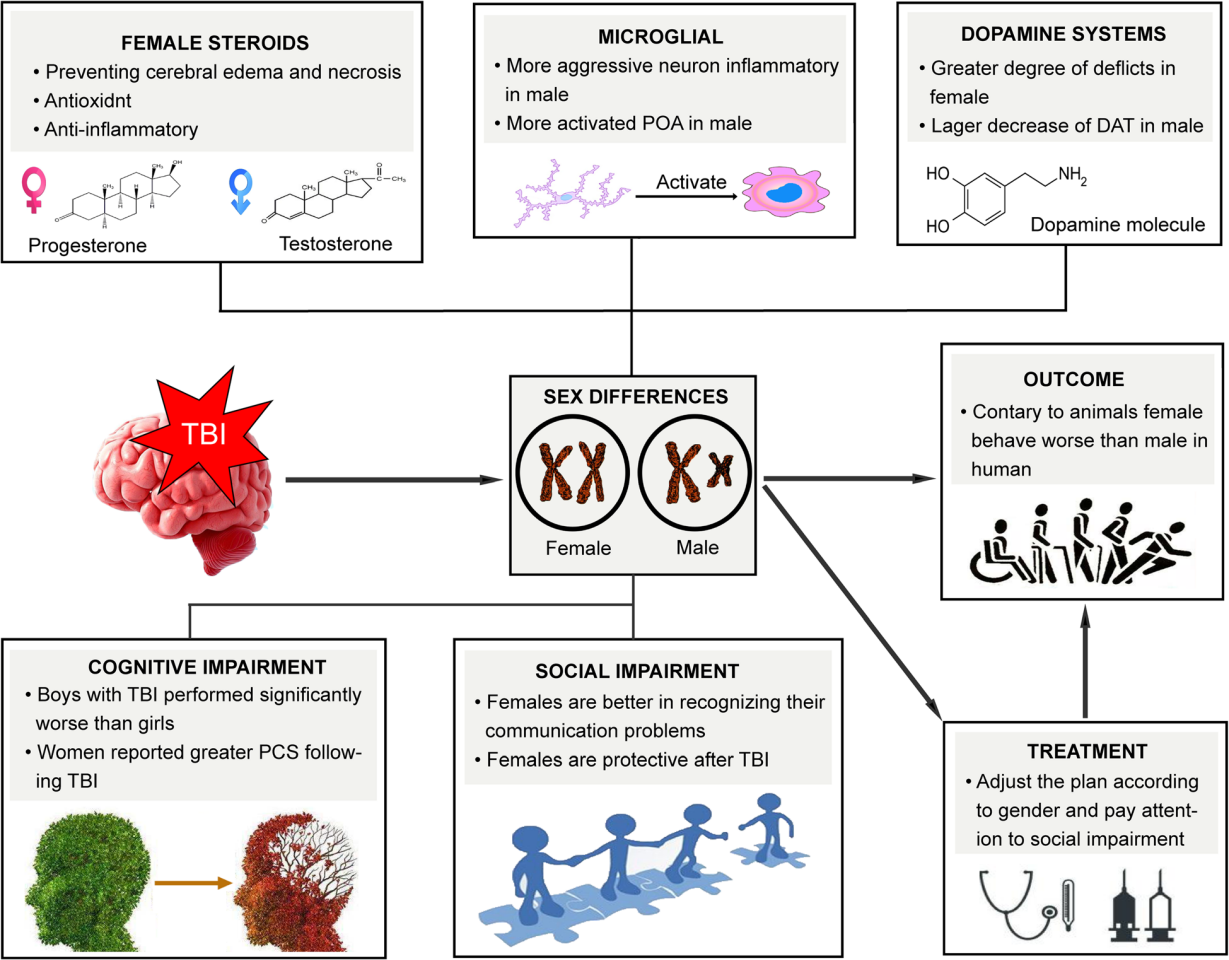
Sex difference after TBI is composed of many factors such as steroids, microglial, and dopamine system. These factors work together and contribute to the outcome

Here, we briefly outlined what is currently known about gender differences in TBI at clinical and pre-clinical levels to serve further research (Fig. 1). We concluded that a variety of factors regulates sex differences in TBI, and it is believed that differences in cognitive impairment after TBI can guide further clinical treatment.

### The fundamental difference

The fundamental difference between male and female originates from the sex chromosome, gender is male or female is determined by the Y chromosome gene, sex chromosomes determine the difference in structure between men and women [12, 13]. Through a series of complicated processes, human beings are physically divided into men and women. When it comes to the brain, there are no apparent structural differences between males and females. However, previous studies have demonstrated minor differences on weight, fine structure, and functional anatomy on human brain across different sexes [14–18]. Since the brain indeed has a “gender,” it is necessary to study and analyze it.

### Sex differences in outcomes after TBI

TBI is a complex process, except for the trauma itself, which brings a series of follow-up problems that lead to the diversity of the outcome. Among the many factors that may influence the prognosis, gender is one of the most controversial. Several studies proved that women have a worse clinical outcome than men after TBI [19–23]. Clinical research including 1627 TBI patients which categorized them into the pediatric group (< 19 years), middle group (19–60 years), and elderly group (> 60 years) by their age found that female TBI group differs in the severity of injury and mortality from male significantly [24]. The mortality rates for male and female were 1.6% and 3.4%, respectively. They also found that the degree of damage measured by the Glasgow Coma Scale (GCS) shows that female performed worse. Besides, female aged 30 years or older had a poorer outcome than either males or females younger than 30 measured by the Glasgow Outcome Scale (GOS) [22].

On the other hand, women tend to have fewer complications after TBI [25], and they usually perform better in prognoses and outcomes [26–30]. Premenopausal women have a better prognosis due increased level of sex hormones [19]. In contrast to the prepubescent female, the pubescent female has lower mortality after isolated moderate-to-severe TBI [31]. In older adults, a study indicated that no sex differences were found in mortality following isolated TBI [32]. An investigator observed 427 TBI patients found no gender difference on Glasgow Outcome Scale while age and initial injury severity had an effect on the GOS [33].

The conclusions of these studies are reversed [32, 34], as TBI outcome is affected by many confounding factors, such as age, severity of TBI, and physical condition of the patient [35–38]. Males are usually disproportionately represented in many studies [31, 39]. The subjects included in the experiment are often males, and the gender differences between male and female are neglected. Therefore, it is necessary to study the gender differences of TBI.

### The effect of female steroids after TBI

As mentioned above, the difference in endogenous hormones may contribute to sex differences after TBI [40]. Steroids such as progesterone and their metabolites protect glial cells and neurons by preventing the brain from edema, necrosis, apoptosis, and inflammation [41–44]. Animal experiments have proven that estrogen and progesterone both play a crucial role in gender differences after TBI [45, 46].

Recent evidence also suggests that estrogen and progesterone levels act as a critical factor in the prognosis of traumatic brain injury. It has been confirmed that the decrease in 17β-estradiol (E2) level leads to an increase in brain damage, while in the proestrus period when the E2 level is high, brain damage is lower [47]. E2 also acts as a neuroprotective factor against stroke in ovariectomized (OVX) animal models [48], which reveals that serum estrogen and progesterone may decrease post-traumatic brain water volume. When applied with pharmacological dose of estrogen and progesterone, male and OVX female rats with TBI model shows a decreased intracranial pressure, improved cerebral perfusion, and increased neurological function score [11].

**Fig. 2:**
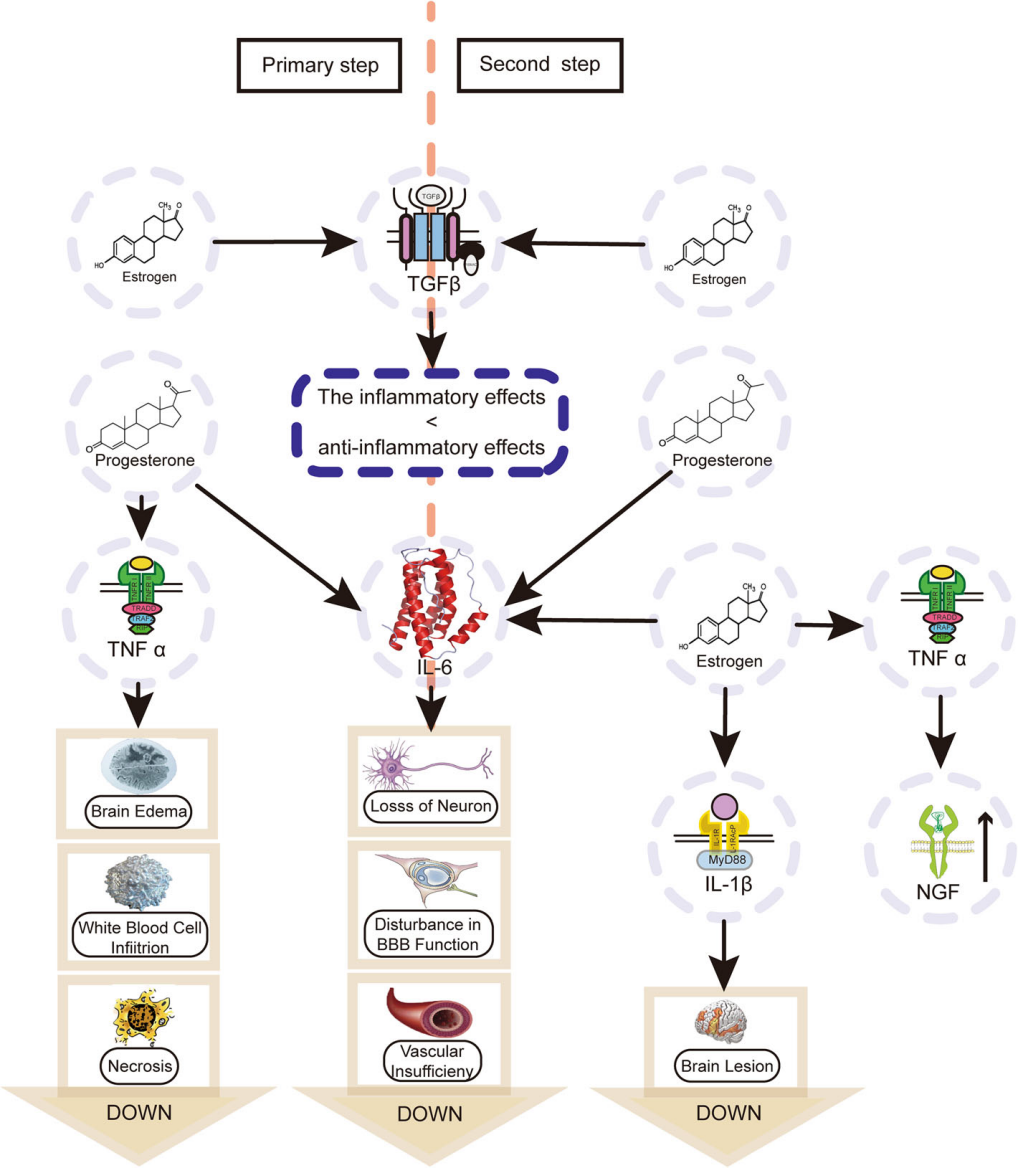
The mechanism of estrogen and progesterone in the acute phase after TBI; the anti-inflammatory effects are greater than inflammatory effects. IL-6 and TNF-α bring anti-inflammatory effects on the brain while TGF-β brings inflammatory. Usually, the former is stronger; thus, estrogen and progesterone benefit from the brain after TBI

Several studies have investigated the possible mechanism of the protective effect of estrogen and progesterone [49]. Thirty minutes after a moderate TBI, hormones or vehicle were intraperitoneally injected, and the levels of proinflammatory cytokines in the brain were measured at 6 and 24 h. The events in the central nervous system after TBI occur in acute and chronic recovery phases, and the former includes a primary and secondary step [50]. At the primary and secondary stage, proinflammatory cytokines such as interleukin-6 (IL-6), interleukin-1 beta (IL-1β), transforming growth factor-beta (TGF-β), and tumor necrosis factor-alpha (TNF-α) are influenced by progesterone and estrogen. Cytokines like IL-1β, IL-6, and TNF-α are confirmed to promote inflammatory responses [51], and increased levels of these proinflammatory cytokines have been observed in the cerebrospinal fluid of brain parenchyma tissues and can intensify the brain lesions that occur during trauma [52, 53]. Thus, the neuroprotective effects of progesterone may be partially caused by a decreased level of anti-inflammatory cytokines including TNF-α at the primary step as well as IL-6 either at the primary or second step after TBI. The neuroprotective effect of estrogen may be partially caused by decreased IL-1β level in the second stage. Besides, higher TNF-α is beneficial in the second step since it can promote the production of the nerve growth factor [54]; thus, estrogen acts as a protective effect in the secondary phase mediated by improving levels of TNF-α (Fig. 2). TGF-β exerts anti-inflammatory effects by inhibiting the levels of IL-1β, TNF-α, and oxygen-free radicals. The anti-inflammatory effect of TGF is higher than that of inflammatory effects. Increased TGF-β is part of the mechanism of anti-inflammatory [55].

Hormone-regulated antioxidant mechanisms are also responsible for gender differences after TBI [19]. The secondary damage after TBI is mainly caused by hypoperfusion of the brain, lipid peroxidation along with the free radical production induced by ischemia [56]. Progesterone treatment in male rats after frontal cortex contusion leads to lower lipid peroxidation [57]. A similar research investigated the level of F2-isoprostane, which is widely used as biomarkers of lipid peroxidation [58]; in 68 TBI patients, they found that male has a higher level of F2-isoprostane than females in CSF [53]. These studies indicate that the neuroprotective effect of progesterone may contribute to its anti-lipid peroxidation function. When it comes to estrogen, it has been shown to have even better antioxidation effects than the most widely used antioxidant supplement vitamin E [59]. Protein carbonylation is used as a marker of the level of oxidative stress in the nervous system because it is a product of the oxidative period [60, 61]. Both female and male rats have a distinct increase in carbonylation after TBI in the injured area. However, when it comes to the field far away from the wounded zone, such as the ependymal tissue of the third ventricle and median eminence, the male has higher carbonylation expression. Considering progesterone also regulates metabolic functions in the nerve system [62] and acts the anti-excitotoxicity effect of TBI, progesterone may play a decisive role in reducing protein carbonization in the female, and the detailed mechanism still needs further research.

### Sex differences in microglia after TBI

Microglia are resident macrophages in the brain and play a key role in brain sexual development via secretion of cytokines and growth factors [63–67]. Although microglia had infiltrated to the brain and started to colonize before there was a distinct gender difference [68], its sexual dimorphism during the development of growth has been detected [69]. In the early postnatal period, microglia are in a primarily activated state [70, 71]. By the end of the third postnatal week, testosterone produced by male testis aromatized into estradiol to induce brain masculinization [72].

Preoptic area (POA) of the hypothalamus is thought to be responsible for thermoregulation and male sexual behavior, and recent studies have indicated that the number and activation state of microglial cells in female rat pups are less than male rat pups in this region [73, 74]. During the brain masculinization, estradiol leads to the upregulation of PGE2, and microglia amplify this signaling pathway to induce the development of masculine dendritic spine phenotype [73]. Similar differences also exist in the hippocampus, amygdala, and parietal cortex [71]. This phenomenon proves the interaction between microglial and sex hormones. Also, it is confirmed that after TBI, the level of activated microglia in the cortex of male rats is far higher than that of female [75]. Microglia of 60-day-old female rats have a higher level of IL-1 and less IL-10 expression compared with male rats [71], while no difference in estrogen receptors was found [76]. With Iba1 immunofluorescence staining, males present higher density near the lesion compared with females after TBI [35].

Moreover, this difference may also link to female steroid hormones on microglia as a pre-clinical study of rat ischemia-reperfusion model found similar levels of microglial activation between male and ovariectomized female mice treated with 17β-estradiol [73, 77]. When inhibiting the microglia, the production of PGE2 is restrained, which means that the microglia affect the production of these hormones [73]. Those hormones can show a neuroprotective function when used after neural injury by reducing the level of reactive microglia, which eventually avoid the overactivated inflammatory period [78]. The view of microglia has transferred from a kind of negative role to positive and protective debris elimination [79].

Existing studies on gender differences of microglia in TBI mainly focus on the interaction of microglia and sex hormones, and studies on other mechanisms of microglia are still insufficient.

### Sex differences in dopamine systems after TBI

To our knowledge, dopamine (DA) systems can mediate multiple aspects of cognition [80], CT scan shows that dopamine transporter (DAT) reduce chronically after TBI [81]. A study involved 24 male and 24 female rats which revealed that compared to females, males have a decrease in DAT expression caused by CCI [82]. Vesicular monoamine transporter-2 (VMAT-2), which is an integral membrane protein transport monoamines, can isolate DA to prevent its oxidation in the cytoplasm and can also sequester neurotoxins in vesicles [83]. Female rats are more susceptible to the inhibition of VMAT-2 after TBI, which leads to a more severe deficit in female rats when the function of DA-storage and VMAT-2 is inhibited after TBI [84].

Estrogen may exert an influence on this process, as it has been proven to regulate DA systems through genomic and nongenomic mechanisms, but the detailed mechanism is still unknown [85]. The extracellular difference happens mainly at the part of dopamine release and synthesis [86]. Females also have a higher density of DAT binding site than males [87–89]. Thus, although the specific mechanism is unclear, there is a particular gender difference in the dopamine system after TBI. A clinical trial included 193 adults of severe TBI observed a significant relationship between sex and DA pathway, which indicates that sex-specific treatment of drugs involved in DA pathway might be a way to improve the overall cognitive recovery for TBI patients in the future [90].

### Sex differences in cognitive impairment after TBI

Cognitive function impairment is a common complication after TBI, which adds a huge burden to the patient’s recovery [8, 19]. In the existing literature, when evaluating cognitive recovery in patients with TBI, men usually have a better recovery on verbal tasks while women can restore their spatial positioning at a faster speed [91–93].

Post-concussive symptoms (PCS) are a set of symptoms that comes after TBI, including loss of memory, difficulty in concentrating, and personality change [94]. Females are more frequently to be reported as patients of PCS compared to male. A meta-analysis pointed out that although men are more frequently to suffer from TBI, worse prognosis is found to be related to female sex [20]. The reason why female patients reported elevated PCS could be partially explained by a risk factor called anxiety sensitivity (AS), which is the tendency to perceive general environmental stimulus as harm or danger [59]. Women have been reported elevated AS both in nonclinical [59] and clinical samples [95]. It is confirmed that patients with higher AS tends to exaggerate PCS symptoms by amplifying its severity [96].

As to the pediatric population, gender can also be a factor that contributes to the different cognitive outcomes after TBI [97]. Boys with TBI performed significantly worse than girls with TBI on the California Verbal Learning Test, which is a neuropsychological test used to evaluate verbal learning and memory capability [98]. Another investigation revealed that boys performed worse than girls and even worse than their counterparts in the control group [99]. This difference is not feasible with sex hormones because the hormone level of male and female is roughly the same before sexual maturity.

In general, due to the complexity of cognitive function, a single clinical test cannot fully assess the difference between male and female in this aspect. Further work needs to be done to establish a throughout understanding of cognitive function changes in patients with TBI.

### Sex differences in social impairment after TBI

After a moderate or severe TBI, patients usually suffer from social impairment when patients trying to return to society [100, 101], because social and behavioral competencies are vulnerable to compromise in brain trauma [102]. Patients may show inappropriate behaviors or loss of social functions [103]. Even children after TBI have a higher risk to be rejected by their friends and lead to long-term social and behavioral problems [104]. Identifying the different social impairment level on each gender may better guide clinical treatment and interventions.

TBI can also lead to language function impairment. Patients after TBI may have difficulty in organizing language and conceiving linguistic stimulus [105, 106]. A research investigated 160 adults with TBI and 81 adults without TBI; by using a standardized measurement of communication problems in everyday life, they found that when communication problems happen, female patients are more accessible to recognize their situations and try to solve them [82]. A late meta-analysis reported that over 39% of patients in the chronic phase after moderate or severe TBI have a significant impairment at recognizing facial affect [107]. The area injured and the severity of the brain may be a risk factor since different parts of the brain have different functions [108]. However, other sources reported that patients with TBI who had damaged the emotion recognition regions did not perform significantly worse than those who had damage in other brain regions [109]. Because plenty of researches have proven the female advantage in recognizing emotion [110], gender may be a potential predictor of emotion recognition deficits. A recent study which tested 53 individuals with TBI and 49 comparisons by both static and dynamic tests found that male patients performed significantly worse not only than female patients but also than comparison participants in the dynamic task [111].

These findings suggested that female gender may play a protective role for social impairment after TBI, although there are opposite results in the animal experiment. Species may cause the differences between human and mice [112] since social communication of animal is much simpler.

### Sex differences in treatment after TBI

Since gender differences exist in the outcome, clinical manifestation, and cognitive impairment after TBI, we have reasons to speculate gender differences in the treatment after TBI. Studying gender differences in treatment after TBI can provide theoretical support for gender-specific treatment at the clinical stage and improve the treatment outcomes. The first idea to break into the brain is to treat with hormones.

As mentioned before, female steroids affect patients through a series of mechanisms. Hormones have begun to be used in the treatment of nervous system damage [113], but it still needs to be adjusted according to gender. Administration of estrogen can increase dendritic spines and improves synaptic connectivity [114, 115]. Tests for post-injury motor function have shown that men have better neurological recovery through estrogen therapy [116]. When male and female are treated with estrogen, female tends to show more adverse effects because the receptors of estrogen of the female are more than those of male, which means that female are more likely to have excitotoxicity and cell death after estrogen therapy. This effect usually counteracts the anti-inflammatory effect of estrogen [117]. Studies also found that estrogen and progesterone are available for TBI treatment. Among them, estrogen is a better choice if used as a prophylactic treatment for women with a high risk of stroke while progesterone is preferred for the male since fewer side effects would happen compared with estrogen [118, 119].

In addition to hormone therapy, other treatments for gender differences of TBI are also under investigation. Related researches have been carried out in animal models. Research using pigs to mimic young and older children TBI found that epinephrine (EPI) can prevent damage to the brain’s autoregulation and necrosis of hippocampal neurons in both newborn and female juvenile pigs after TBI [120–122]. Phenylephrine (Phe) also had been found to reduce the damage of the K channel, which can lead to the impairment of cerebral and brain regulation function in female [123].

Hypothermia is a new type of treatment that has long been indicated but is not widely used in clinical practice due to its reversal study findings in both animal models and human trials in brain injury [124–126]. In recent years, investigators found that gender may affect the efficacy of hypothermia [127–129]. In a study of treating TBI rats with hypothermia, post-traumatic hypothermia significantly reduced more overall contusion volume in males than females and protected cortical neurons in males but no effect in females [8].

## Conclusion

Gender has an impact on many aspects of TBI, including clinical manifestations, cognitive impairments, and outcomes, which can be used to develop sex-specific treatment and improve prognosis. This review explores the causal differences in clinical signs, cognitive impairments, medications, and prognosis between males and females after TBI in different dimensions from the view of genes, hormones, cells, individuals, and society. As it turns out that multiple factors contribute to the gender differences after TBI, not merely the perspective of gender and sex hormones. The reason for differences in mortality between male and female after TBI remains inconclusive, but lots of researches have proven that women outperform men in cognitive from cognitive function impairment. Because of these differences between male and female, precise treatment should be taken based on gender. More researches are needed to figure out the entire mechanism of sex difference in TBI, since most studies only focused on the effect of sex hormones. Besides, more attention is needed to compensate for the lack of previous studies which only incorporated into male animals, and clinical studies should not neglect gender differences to avoid bias in real experimental results.

## Data Availability

All data generated or analyzed during this study are included in this review.

## Abbreviations

AS: Anxiety sensitivity
CPP: Cerebral perfusion pressure
DA: Dopamine
DAT: Dopamine transporter
E2: 17β-estradiol
EPI: Epinephrine
GCS: Glasgow Coma Scale
GOS: Glasgow Outcome Scale
ICP: Intracranial pressure
IL-1β: Interleukin-1 beta
IL-6: Interleukin-6
OVX: Ovariectomized
PCS: Post-concussive symptoms
Phe: Phenylephrine
POA: Preoptic area
TBI: Traumatic brain injury
TGF-β: Transforming growth factor-beta
TNF-α: Tumor necrosis factor-alpha
VMAT-2: Vesicular monoamine transporter-2
